# Contributions of Luminance and Motion to Visual Escape and Habituation in Larval Zebrafish

**DOI:** 10.3389/fncir.2021.748535

**Published:** 2021-10-21

**Authors:** Tessa Mancienne, Emmanuel Marquez-Legorreta, Maya Wilde, Marielle Piber, Itia Favre-Bulle, Gilles Vanwalleghem, Ethan K. Scott

**Affiliations:** ^1^The Queensland Brain Institute, The University of Queensland, Saint Lucia, QLD, Australia; ^2^School of Medicine, Medical Sciences, and Nutrition, University of Aberdeen, Aberdeen, United Kingdom; ^3^School of Mathematics and Physics, The University of Queensland, Saint Lucia, QLD, Australia

**Keywords:** zebrafish, vision, habituation, calcium imaging, light-sheet fluorescence microscopy, predator-prey, tectum, superior colliculus

## Abstract

Animals from insects to humans perform visual escape behavior in response to looming stimuli, and these responses habituate if looms are presented repeatedly without consequence. While the basic visual processing and motor pathways involved in this behavior have been described, many of the nuances of predator perception and sensorimotor gating have not. Here, we have performed both behavioral analyses and brain-wide cellular-resolution calcium imaging in larval zebrafish while presenting them with visual loom stimuli or stimuli that selectively deliver either the movement or the dimming properties of full loom stimuli. Behaviorally, we find that, while responses to repeated loom stimuli habituate, no such habituation occurs when repeated movement stimuli (in the absence of luminance changes) are presented. Dim stimuli seldom elicit escape responses, and therefore cannot habituate. Neither repeated movement stimuli nor repeated dimming stimuli habituate the responses to subsequent full loom stimuli, suggesting that full looms are required for habituation. Our calcium imaging reveals that motion-sensitive neurons are abundant in the brain, that dim-sensitive neurons are present but more rare, and that neurons responsive to both stimuli (and to full loom stimuli) are concentrated in the tectum. Neurons selective to full loom stimuli (but not to movement or dimming) were not evident. Finally, we explored whether movement- or dim-sensitive neurons have characteristic response profiles during habituation to full looms. Such functional links between baseline responsiveness and habituation rate could suggest a specific role in the brain-wide habituation network, but no such relationships were found in our data. Overall, our results suggest that, while both movement- and dim-sensitive neurons contribute to predator escape behavior, neither plays a specific role in brain-wide visual habituation networks or in behavioral habituation.

## Introduction

For prey animals, avoiding approaching predators is crucial to survival, but unnecessary escape behavior is both energetically costly and disruptive to normal behavioral routines. As a consequence, there has been strong selective pressure to escape from genuinely threatening stimuli while ignoring innocuous stimuli and stimuli that occur repetitively without consequence. In larval zebrafish, input from the auditory (Burgess and Granato, 2007; Marques et al., 2018), somatosensory (Eaton et al., 1977; Douglass et al., 2008), and lateral line systems (McHenry et al., 2009) can all drive startle responses, but vision, which is uniquely informative about an incoming object’s spatial characteristics, has received particular attention in recent years.

In an experimental context, predators can be simulated on a screen with looming stimuli (looms): dark shapes that grow rapidly from a single point (Luca and Gerlai, 2012). If the loom is sufficiently salient, it elicits a stereotyped escape response, which in larval zebrafish takes the form of a rapid body bend followed by a powerful swim sequence (Dunn et al., 2016; Bhattacharyya et al., 2017; Marques et al., 2018). The core sensorimotor circuitry responsible for visual escape in larval zebrafish has been described (reviewed in Marquez-Legorreta et al., 2020), involving the perception of motion and luminance by specialized retinal ganglion cells (RGCs) (Temizer et al., 2015), widespread and powerful loom responses in neurons belonging to the tectum (called the superior colliculus in mammals) contralateral to the stimulus (Helmbrecht et al., 2018), and downstream activation of reticulospinal neurons in the hindbrain (Sato et al., 2007; Yao et al., 2016). However, many details about how particular components of the loom stimulus are detected and integrated, and how behaviors are gated, remain mysterious.

A dark looming stimulus comprises two components: a decrease in luminance and expanding moving edges at the periphery. These components can be separated experimentally with the presentation of a dimming circle (providing decreased luminance without moving edges) or an expanding isoluminant checkerboard on a gray background (moving edges without a change in overall luminance). Behavioral experiments have shown that expanding checkerboards, and therefore moving edges, are sufficient to elicit escape responses, albeit at a lower rate than dark loom stimuli (Dunn et al., 2016; Heap L. A. L. et al., 2018). The neural circuitry detecting these moving edges is incompletely understood, but it has been shown that specific laminae of the tectal neuropil receive edge-specific information, and that RGCs targeting these specific neuropil layers appear to project exclusively to the tectum and not to other retinal arborization fields (Robles et al., 2014; Temizer et al., 2015). On the other hand, the dimming component involves a retino-thalamo-tectal circuit, which increases the probability of an escape response (though dimming alone elicits startles infrequently), and also determines the direction of this startle (Temizer et al., 2015; Heap L. A. L. et al., 2018). These observations suggest that different components of the looming stimulus are processed in distinct pathways and mediate different facets of the behavioral response.

Habituation is a form of non-associative learning that is important for survival, as it minimizes energy expenditure and disruption from unnecessary responses to repetitive stimuli (Thompson and Spencer, 1966; Rankin et al., 2009). The visual escape response and O-bends habituate in larval zebrafish, with the startle probability decreasing with repeated presentations of loom (Marquez-Legorreta et al., 2019) or dark flash (Randlett et al., 2019) stimuli. It has been proposed that this habituation results from the tectum’s “uncoupling” the perceptual visual circuits in the retina and thalamus from premotor circuitry in the hindbrain, thus shifting sensorimotor gating toward non-responsiveness (Marquez-Legorreta et al., 2019). The tectum is well positioned to integrate movement and luminance information, and has been shown to contain populations of neurons whose responses habituate in parallel with behavioral habituation (Marquez-Legorreta et al., 2019). Nonetheless, the neural circuitry responsible for visual habituation, and the specific ways in which luminance and movement information interact in these circuits, remain incompletely understood.

In this study, we have used light-sheet microscopy and genetically encoded calcium indicators to track activity in individual neurons across the brains of larval zebrafish while presenting repetitive loom, checkerboard, and dimming stimuli. The goal has been to describe each neuron’s anatomical position; selective responsiveness to movement, luminance, and full loom stimuli; and degree of habituation to repeated stimuli. By providing links between these anatomical and functional readouts, we have aimed to determine the stimulus properties and brain regions responsible for behavioral habituation to repetitive threatening visual stimuli. Behaviorally, we have found that the visual escape response of zebrafish larvae does not habituate to checkerboard stimuli, and that the habituation to looms requires both the movement and luminance components. We have identified the neuronal substrates for the visual habituation to luminance, movement, and looms, and show that loom habituation at the circuit level also requires both components. We have not identified neurons responding only to full looms, suggesting that the integration of the two components drives the sensorimotor gating.

## Materials and Methods

### Animals

All experiments were conducted in accordance with the University of Queensland Animal Welfare Unit’s ethics approval number SBS/341/19. Larval zebrafish of the tupel longfin nacre (TLN) strain were used at 6 days post fertilization (dpf). Larvae were raised in E3 media with methylene blue in 10 cm diameter petri dishes at a density of 50 larvae per dish. Fish used for SPIM experiments expressed the transgene *elavl3:H2B-GCaMP6s* for pan-neuronal nuclear expression of calcium indicator GCaMP6s (Chen et al., 2013).

### Behavioral Experiments

Larvae were loaded individually into circular wells of 15 mm diameter and about 5 mm depth in a 12 well agar mold on a plastic petri dish lid. Wells were filled with E3 media without methylene blue, and the setup was placed 10 mm above an LED screen (Little Bird, Australia, Marquez-Legorreta et al., 2019). The setup was in a dark cabinet, and a lens (40 mm Thorlabs) and a 665 nm longpass filter (FGL665 - Ø25 mm RG665 Colored Glass Filter, Thorlabs) delivered infrared light to the camera with a weak signal from the screen that confirmed the timing of the looming stimuli. Fish movement was recorded from above with a Basler acA1920 camera at 30 frames per second. Movements were tracked and binned into 1 s increments with Viewpoint software (ZebraLab, ViewPoint Life Sciences, France), and classified into three speed groups: <0.5 mm/s (drift), 0.5–30 mm/s (scoots), and >30 mm/s (escapes). Fish that did not show any escapes during the experiment or were not trackable by the Viewpoint software were excluded. The stimulus train consisted of 5 min without stimuli (gray background: hsl 0% 0% 85%), then 20 presentations of either a black loom, checkerboard loom, or dim, followed by 10 black looms. The black loom expanded from a single point in the center of the well, and reached a maximum angle of expansion of approximately 90°. The checkerboard loom was made up of white and black squares at such a ratio to be isoluminant with the background gray [as measured by a Digital Lux meter (LX1010B)]. The dimming stimulus began at the background luminance and decreased to black (hsl 0% 0% 0%). All stimuli progressed in a hyperbolic manner and reached their maximum state (width of 25 mm for the looms, full darkness for the dims) 5 s after onset. Each stimulus then faded back to the background gray in a linear manner over 15 s. The inter-stimulus interval was variable (20, 25, 30 or 35 s).

The probability of an escape response was calculated as the proportion of larvae that performed an escape in response to a stimulus. Using R software, the nparLD package (Noguchi et al., 2012) was used to test for habituation within each group by comparing responses to the first and last stimuli of the initial block of 20 stimuli, and the first stimulus of the loom block. A Mann-Whitney U test was used to compare responses between the different stimuli trains, with a Bonferroni correction for the multiple comparisons. The fitted curves were produced in GraphPad Prism v 9.1.1 with the exponential one phase decay curve from the 1st to the 20th stimuli of each block, using a Least Squares regression and plateau to 0, and a simple linear regression from the 21st to the 30th stimuli.

### Calcium Imaging Experiments

Larvae were mounted in 2% low melting point agarose (Sigma, A9045) in a custom cubic 3D-printed chamber (24 mm width, 24 mm length, 20 mm height), with glass coverslips forming the four walls (Favre-Bulle et al., 2018, 2020). The chamber was filled with E3 media (without methylene blue) after agarose was set. Larvae were imaged using a custom-built light-sheet microscope (Taylor et al., 2018). A single light sheet illuminated the fish from the rostral direction, and the width of the sheet was kept narrow enough (laterally) to minimize the amount of light entering the eyes (Constantin et al., 2020). A total of 50 z-planes over a range of 250 μm dorso-ventrally were imaged to capture data from the full volume of the brain at a rate of 2 Hz. Images were binned 4x to a final resolution of 640 × 540 pixels, in tagged image file format (TIFF).

### Loom Stimulus Trains for Calcium Imaging

Stimuli were presented on a 75 mm × 55 mm LCD generic PnP monitor (1,024 × 768 pixels, 85 Hz, 32-bit true color) with an NVIDIA GeForce GTX 970 graphics card (Thompson and Scott, 2016; Thompson et al., 2016). The monitor was positioned 30 mm to the right or left of the larvae, and was covered by a colored-glass alternative filter (Newport, 65CGA-550) with a cut-on wavelength of 550 nm. The maximum angle the loom covered was approximately 86°. Stimuli had the same luminance and expansion dynamics as in the behavioral experiments. All stimulus trains began with 30 s of baseline imaging before the first stimulus onset and finished after the last stimulus finished fading back to background levels. Data for each of the three calcium imaging experiments were collected from different cohorts of larvae. The stimulus train for the first experiment consisted of 10 presentations of either dims, checkerboards, or black looms, followed by five black looms, with an interstimulus interval of 20 s. For the second experiment, the stimulus train consisted of a single dim, then a single checkerboard, then a single loom, spaced apart by 1 min, followed by the same sequence again after a 5-min break. In this dataset, stimuli were presented to the left instead of the right eye. For the third experiment the stimulus train consisted of a dim, checkerboard, dim, checkerboard block, followed by 10 looms, then a repetition of the initial block. This experiment again used a 20 s inter-stimulus interval.

### Extraction of Fluorescent Traces of Calcium Activity

Calcium imaging data were reformatted into separate TIFF files for individual z-planes (50 planes per fish) in ImageJ v1.52c. Motion correction was performed using the Non-Rigid Motion Correction (NoRMCorre) algorithm, and fluorescence traces were extracted and de-mixed from the time series using the CaImAn package (version 0.9, ^1^) (Pnevmatikakis et al., 2016; Pnevmatikakis and Giovannucci, 2017; Giovannucci et al., 2019). We used 4,000 components per slice to ensure that we would not miss any ROIs during the initialization step of CaImAn. The risk of over-segmentation was mitigated by a merge step using a threshold of 0.8 to merge overlapping ROIs. The order of the autoregressive model was set at 1 to account for the decay of the fluorescence, our acquisition speed being too slow to account for the rise time. The gSig (half-size of neurons) was set at 2, based on estimates of the sizes of the nuclei in our images. We did not use any temporal or spatial down-sampling and the initialization method was “greedy_ROI.” The components were updated before and after the merge steps, empty components were discarded, and the components were ranked for fitness as previously (Pnevmatikakis et al., 2016). After the fluorescence traces were extracted, the filtered noise was added back to each ROI to account for possible negative signals (Vanwalleghem et al., 2021).

### Analysis of Whole Brain Calcium Activity

For each of the three datasets, fluorescent traces from all of the ROIs found by CalmAn were pooled and Z-scored. In the first calcium imaging dataset, consisting of the three different stimulus trains where 10 stimuli (dims, checkerboards, or looms) were first presented followed by five looms, a linear regression to the first 10 stimuli allowed us to identify ROIs responsive to their respective component. ROIs with an r^2^ value greater than the median + 2SD (=0.1158) were used for subsequent analyses. The maximum response of these ROIs to each stimulus was calculated as the maximum z-score value within the window of the stimulus presentation minus the baseline prior to the presentation. These were then averaged per fish before they were averaged per stimulus train. Using R software, the nparLD package (Noguchi et al., 2012) was used to test for habituation within each group by comparing the average maximum responses to the first and last stimuli of the initial block of 10 stimuli. A Mann–Whitney *U* test was then used to compare responses between the different stimuli trains, with a Bonferroni correction to adjust for the multiple comparisons.

Our second dataset consisted of a sequence of dims, checkerboards, and looms repeated twice. Applying linear regression to each pair of stimuli identified visually responsive ROIs, and those with a r^2^ value greater than the median + 2SD (=0.3164) were used for subsequent analyses. This included a K-means clustering with 10 clusters and 20 replicates. This number of clusters was set to ensure over-clustering in order to allow removal of response profiles which were not common across all animals. In order to be included in subsequent analyses, clusters were validated by checking for consistency: the correlation of each ROI within the cluster to the cluster mean had to be greater than 0.5, the cluster had to contain at least 100 ROIs, and be represented in at least 75% of fish, with no more than 33% of ROIs contributed from any single fish. These exclusion criteria yielded five clusters. Three of these clusters were then manually combined based on similar checkerboard-selective response profiles (Supplementary Figure 1), resulting in three major clusters. We then calculated the average proportion of ROIs across fish that were visually responsive in each brain region, by dividing the number of ROIs that passed the aforementioned criteria by the total number of ROIs identified in that brain region for each fish. Next, we did the same calculations to find the average proportion of ROIs within each brain region belonging to each of the three major clusters above, and then normalized them.

Lastly, the third dataset consisted of a sequence of dim, checkerboard, dim, checkerboard, followed by 10 loom repeats and finishing with another sequence of dim, checkerboard, dim, checkerboard. We applied a linear regression to the first and last four stimuli, and again, ROIs with r^2^ value greater than the median + 2SD (=0.1256) were kept for subsequent analyses. For these selected ROIs, the maximum response to each stimulus presentation was calculated by identifying the maximum z-score value within a window of that stimulus presentation adjusted by the baseline just prior. To classify ROIs sensitive to dims, checkerboards, or both components, we used the criteria described in Supplementary Table 1. Next, to look at the different rates of habituation within these groups, an exponential decay curve (*f*(*x*) = *a* + *be*^−cx^) was fitted to the maximum response to the 10 looms for every ROI. We used the robust least absolute residual regression function of MATLAB to limit the impact of outliers. To look at the rates of decay, we validated the goodness of fit by taking ROIs that had an adjusted r^2^ value greater than 0.5 and a sum of squares due to error less than 20. A Friedman Test was performed to test for differences between the median of c values for each group within each fish.

### Registration to Reference Atlas for Anatomical Classification

We used Advanced Normalization Tools (ANTs, ^2^) to register the ROIs to the H2B-RFP reference of Zbrain. Images from the SPIM experiments were warped to a common template previously acquired and registered to the Zbrain atlas. The resulting warping parameters were applied to the xyz coordinates of the centroids of the ROIs to map them into the 294 brain regions defined in the Zbrain atlas (Randlett et al., 2015; Poulsen et al., 2021).

### Data Visualization

For visualizing the ROIs, we used Unity to represent each as a sphere and map it back to the brain. An isosurface mesh of the zebrafish brain was generated from the Zbrain masks for the diencephalon, mesencephalon, rhombencephalon, telencephalon and eyes using ImageVis3D71 (Poulsen et al., 2021). The mesh was imported in Unity and overlaid to the ROIs. Thus allowing a 3D spatial visualization of the ROIs distributions encountered. Distributions of ROIs are shown in the main figures as the combined registered ROIs from all animals in the experiment. We represent them this way because the distributions were highly consistent from animal to animal (Supplementary Figure 2).

## Results

It has previously been shown that drops in luminance (dim), moving edges (checkerboard), and loom (combining dim and edges) stimuli lead to different types and probabilities of visual startle behavior in zebrafish larvae (Dunn et al., 2016; Heap L. A. L. et al., 2018), and that startle behavior habituates in response to repeated loom stimuli (Marquez-Legorreta et al., 2019). This leaves two open questions about the relationships between visual stimulus components and habituation. First, it is unknown whether behavioral responses to repeated dimming or checkerboard stimuli habituate, as they do in response to repeated loom stimuli. Second, it is unclear whether prior exposure to repeated dims or checkerboard stimuli is sufficient to habituate animals to loom stimuli.

**FIGURE 1:**
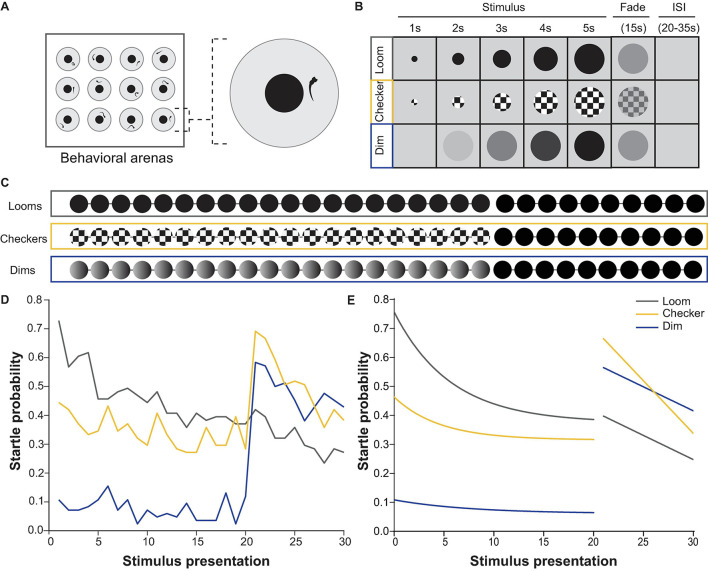
Behavioral responses to repeated loom, checkerboard, and dim stimuli. **(A)** Experimental set up used for behavioral experiments, where 12 larvae were presented with stimulus trains from a screen below while their movements were recorded with a camera above. **(B)** Properties and timing of the loom, checkerboard, and dim stimuli. **(C)** The three stimulus trains used for behavioral experiments, comprising 20 presentations of one stimulus (either loom, checkerboard, or dim) followed by 10 looms, all separated by interstimulus intervals ranging between 20–35 s. **(D)** The average probability of an escape response during these experiments, across all larvae. Habituation was significant (*p* = 4.28E-7) for loom responses, but not for checkerboard (*p* = 0.01325535) or dim (*p* = 0.8195796, tested with the nparLD R package, using Bonferroni correction for multiple comparisons, adjusting the *p*-value for significance to 0.00625). Responses to the first loom (21st stimulus) were not significantly different from naive loom responses for animals shown checkerboards (*p* = 0.4875) or dims (*p* = 0.05995, tested with a Mann–Whitney *U* test, using Bonferroni correction for multiple comparisons, adjusting the *p*-value for significance to 0.00625). **(E)** Fitted curves to the average probability of escape response shown in panel **(D)** illustrate the habituation profile to each stimulus type.

To address both questions, we designed a behavioral apparatus (Figure 1A) and stimulus train (Figures 1B,C) in which larvae were presented with 30 visual stimuli in close succession (20–35 s interstimulus interval, ISI). The first 20 stimuli were either checkerboards or dims, followed by 10 looms. A third group of larvae was shown 30 looms. As previously described, loom stimuli initially caused a high rate of visual startle behavior (Temizer et al., 2015; Dunn et al., 2016; Heap L. A. L. et al., 2018) and these responses gradually habituated to repeated loom stimuli (Figure 1D; Marquez-Legorreta et al., 2019). By comparison, larvae startled to looming checkerboards at a lower rate, and these responses did not undergo significant habituation over the course of 20 trials. Dimming stimuli seldom drove escape behavior, and therefore could not be assessed for habituation. When 20 trials of either dims or checkerboards preceded loom trials, no significant reduction was seen in the responses to the first loom (the 21st trial), as compared to naïve animals seeing a loom stimulus as their first trials (Figure 1E). These results provide no evidence for behavioral habituation to checkerboard stimuli, and suggest that the circuits mediating loom habituation may depend on both components of the loom stimuli, rather than either moving edges or dimming in isolation.

**FIGURE 2:**
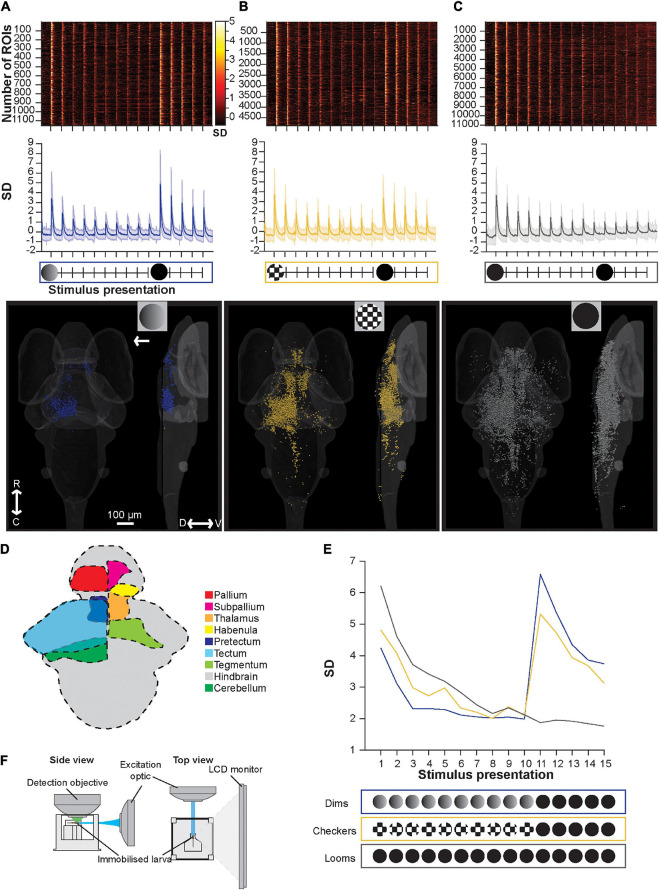
Brain-wide calcium responses to repeated loom, checkerboard, and dim stimuli. **(A)** A raster plot (top), average trace with S.D. shaded (middle), and transverse and lateral spatial distributions (bottom) of dim sensitive ROIs, arrow represents position of stimulus presentation. **(B)** Shows the same information for ROIs sensitive to checkerboards, and panel **(C)** shows this information for ROIs responsive to looms. **(D)** Schematic representation of key regions’ locations in the larval zebrafish brain. **(E)** The average maximum brain-wide response of dim, checkerboard, and loom sensitive ROIs and the stimulus trains used during these calcium imaging experiments. Habituation was significant (*p* = 3.42E-18) for loom sensitive ROIs, checkerboard sensitive ROIs (*p* = 1.91E-8) and dim sensitive ROIs (*p* = 2.43E-4, tested with the nparLD R package, using Bonferroni correction for multiple comparisons, adjusting the *p*-value for significance to 0.01). Responses to the first loom (11th stimulus) were not significantly different from naive loom responses for animals shown checkerboards (*p* = 0.1513) or dims (*p* = 0.4491, tested with a Mann–Whitney *U* test, using Bonferroni correction for multiple comparisons, adjusting the *p*-value for significance to 0.01). **(F)** Experimental set up used for calcium imaging experiments, where larval zebrafish are presented the stimulus train on an LED monitor. R, rostral; C, caudal; D, dorsal; V, ventral.

To probe the functional structure of the circuits underlying these behavioral effects, we showed the equivalent stimulus trains during whole-brain calcium imaging of larvae expressing nuclear-targeted GCaMP6s in all neurons, using a custom-built light-sheet microscope (Figure 2F, see section “Materials and Methods”). We collected volumetric data using 50 planes spanning the brain at 5 μm intervals across the dorso-ventral axis, and tracked activity in individual regions of interest (ROIs) generally corresponding to individual neurons.

In contrast to the behavioral results, we see marked habituation in the responses of dim- and edge-sensitive ROIs during repeated stimulus presentations (Figure 2E). Brain-wide loom-responsive ROIs also undergo pronounced habituation (Figure 2E), in line with startle behavior (Figure 1C). In terms of their abundance and distribution, we identified relatively few dim-sensitive ROIs (1,154 ROIs across 12 animals), and these were primarily in the tectum (Figure 2A). Checkerboard-responsive ROIs were more numerous (4,873 ROIs, 11 animals), and were spread broadly across several brain regions (Figure 2B). Loom stimuli elicited the most numerous (11,050 ROIs, 11 animals) and widespread responses, with an overall distribution resembling that of checkerboard-responsive ROIs (Figure 2C).

There are several interpretations to be drawn from these results. First, we see habituation, as measured by brain-wide calcium responses, to repeated presentations of both dimming and checkerboard stimuli. This is difficult to register to behavior in the case of dim-responsive ROIs, since dimming stimuli seldom trigger escape behavior even in naïve larvae, but it suggests that for motion stimuli, animals sustain behavioral sensitivity to moving edges even as motion-sensitive ROIs (at least as a population) decrease their response strength. It is worth noting the caveat, however, that the stimuli are presented in different orientations (from the bottom for behavioral experiments and from the side during calcium imaging) due to the spatial constraints of the two setups. As has been shown previously (Marquez-Legorreta et al., 2019), brain-wide responses to loom stimuli habituate, paralleling behavioral habituation to looms. Also paralleling our behavioral results (Figure 1), these results provide no evidence of pre-habituation of full loom responses by either checkerboards or dim stimuli, suggesting that only full loom stimuli can cause habituation in the visual escape circuit.

In terms of saliency, at least as judged by the number of brain-wide responses, we find many more and more widespread checkerboard-responsive ROIs than dim-responsive ROIs. Loom responses are more numerous than either, and indeed outnumber the combined dim- and checkerboard-responsive ROIs from the other stimulus trains. This suggests that motion may be the more salient component of the loom, but that there may also be neurons that respond only when a full loom stimulus, with both dimming and motion components, is present.

**FIGURE 3:**
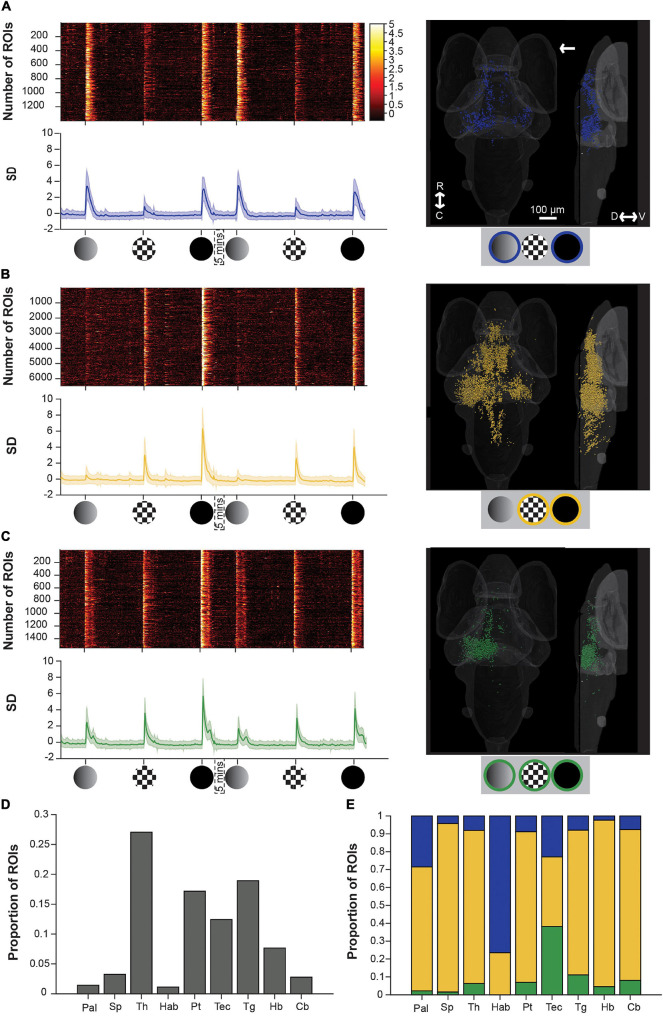
Brain-wide calcium responses to dim, checkerboard, and loom stimuli. **(A)** A raster plot (top left), average trace with S.D. shaded (bottom left) and transverse and lateral spatial distributions (right) of ROIs responsive to dim and loom stimuli. Response strengths are shown in S.D., and the arrow shows the position of the stimulus presentation. **(B)** Shows the same information for ROIs responsive to checkerboards and looms, and panel **(C)** shows this information for ROIs responsive to all three stimuli. **(D)** Average proportion of ROIs within each brain region that are visually responsive. **(E)** Average proportion of visually responsive ROIs within each brain region that are either dim sensitive (blue), checkerboard sensitive (yellow), or sensitive to both dim and checkerboard (green). R, rostral; C, caudal; D, dorsal; V, ventral; Pal, pallium; Sp, subpallium; Th, thalamus; Hab, habenula; Pt, pretectum; Tec, tectum; Tg, tegmentum; Hb, hindbrain; Cb, cerebellum.

Because one habituation experiment would likely impact subsequent ones, the stimulus trains used in Figure 2 were played separately, and each animal only observed one of the trains. This, in turn, prevents us from judging how much overlap there is among dim-, checkerboard-, and loom-responsive ROIs. The specificity or overlap of these responses, however, have important implications for the circuit-level processing underlying network-wide and behavioral habituation. In order to determine the degree to which neurons respond to more than one of these stimuli, while minimizing the impacts of habituation, we presented all three stimuli to the same animals with only two repetitions of each stimulus, and with long intervals between stimuli (Figure 3).

To address the degree to which individual ROIs respond to dim, checkerboard, and/or full loom stimuli, we then used a k-means approach (see section “Materials and Methods”) to identify functional categories of ROIs with distinct response properties to each of the stimuli. This yielded three clusters that were responsive to (1) dims and full looms (but not checkerboards), (2) to checkerboards and full looms (but not dims), and (3) to all three stimuli.

A dim-sensitive cluster (blue, Figure 3A), responsive both to dims and full looms, contained the fewest ROIs, and these were principally spread across the habenulae, thalamus, tectum, and pallium, consistent with previous observations (Cheng et al., 2017; Zhang et al., 2017; Chen et al., 2018; Heap L. A. L. et al., 2018; Robles et al., 2021). On average, these ROIs also had very weak responses to checkerboard stimuli. Another cluster, sensitive to the moving edge component of the loom stimulus (yellow, Figure 3B), comprised checkerboard- and loom-responsive ROIs. Among our three clusters, this was the most abundant in terms of ROI number, and contributed a majority of the ROIs in each of the brain regions that we studied except for the tectum (balanced among the three clusters), and the habenulae (with mostly dim-sensitive ROIs). The final cluster (green, Figure 3C) comprising ROIs sensitive to both dims and checkerboards (and unsurprisingly to looms), was principally contained within the tectum.

These results have a few notable implications. First, neurons responding to moving edges (from checkerboard and full loom stimuli) are much more numerous and broadly distributed in the brain than the luminance signals (from the dim and full loom stimuli), suggesting a rather targeted perception and use of dimming information, at least as manifested by this particular set of stimuli. Second, neurons responding to all three stimuli are numerous in, and largely restricted to, the tectum contralateral to the stimulus (the right tectum in Figure 3). This reinforces the idea that the tectum (superior colliculus) is a key structure in loom perception and likely in visual escape behavior.

However, it is interesting to note that the checkerboard specific responses, and the dim responses to a lesser extent, show a bilateral distribution. This is especially noticeable in the thalamus and tectum, suggesting a position-independent representation of the stimuli. Finally, we did not identify a loom-specific cluster (responsive to looms but not to dims or checkerboards, see Supplementary Methods and Supplementary Figure 3). This suggests that the dim- and checkerboard-responsive ROIs’ responses (Figure 3C) represent the summed inputs of the luminance and movement components of the loom stimulus, rather an emergent signal specific to looms. This notion of the linear summation of the luminance and movement signals is supported by the form of the loom responses in these ROIs, which combines the short, broad double peak seen to dimming stimuli with the tall, sharp single peak elicited by a looming checkerboard (Figure 3C).

**FIGURE 4:**
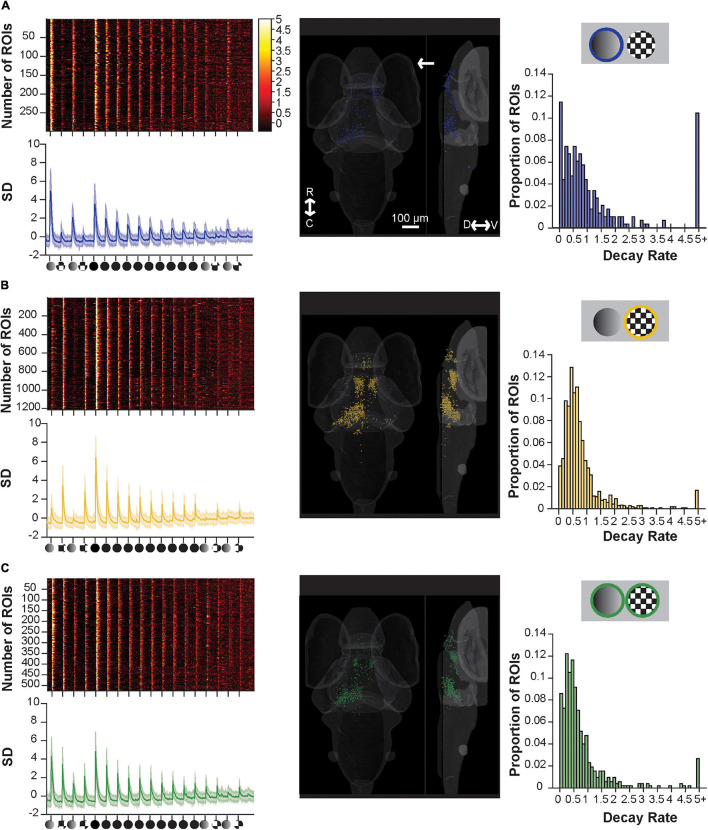
Component sensitive responses and their associated rates of habituation. **(A)** A raster plot (top left), average trace with S.D. shaded (bottom left), transverse and lateral spatial distributions (middle), and histogram of their rate of decay as modeled from fitting with an exponential decay curve (right) for ROIs responsive to dim and full loom stimuli. ROIs with responses ≥5.0 are pooled. Response strengths are shown in S.D., and the arrow shows the position of the stimulus presentation. **(B)** Showing the same information as above but for ROIs responsive to checkerboards and full loom stimuli, **(C)** same as above but for ROIs responsive to dims, checkerboards and full loom stimuli. R, rostral; C, caudal; D, dorsal; V, ventral.

Comparing the spatial distributions of dim- and motion-sensitive ROIs (Figures 3A,B) to the distributions of moderately- and fast-habituating ROIs during repeated looms (as seen in Marquez-Legorreta et al., 2019) suggests possible links between these populations (Supplementary Figure 4). Specifically, fast-habituating ROIs are distributed similarly to those responding to motion, while moderately habituating ROIs’ distribution resembles that of dim-sensitive ROIs. To test whether these are, indeed, the same populations, we devised a stimulus train in which we could identify each ROI’s inherent responsiveness to dims and motion, and its habituation rate to repeated looms (Figure 4). This stimulus train begins with two dims and two checkerboard looms at the beginning of the train, followed by ten loom stimuli, and finishes with two more dims and checkerboard stimuli. This experiment permits us to categorize ROIs by their responsiveness to dims and checkerboards (using the first four trials, analogous to the analysis done in Figure 3), and separately to gauge their habituation rate using the ten looms, as previously performed (Marquez-Legorreta et al., 2019).

By selectively categorizing responses using customized set of criteria (see Supplementary Table 1), we identified functional clusters of ROIs that were introduced in Figure 3: dim- and loom- responsive (Figure 4A), checkerboard- and loom-responsive (Figure 4B), and ROIs responsive to all three stimuli (Figure 4C). Categorizing the responsive ROIs by their inherent stimulus sensitivity then allowed us to look for differences among these groups in their basic responses to looms and in their degree and rate of habituation to repeated loom stimuli. We approached this by fitting an exponential habituation curve to each ROI and using this curve to assess its rate of habituation. We then looked at the statistics of habituation rate across clusters to determine whether these rates differ across clusters.

We found that the rate of habituation during the 10x loom portion of the stimulus train (as judged by the decay constant (*c* in our exponential equation) was similar across all three groups (dim-sensitive median 0.6566, SD 0.2945; checkerboard-sensitive median 0.5794, SD 0.2012; sensitive to both median 0.6695, SD 0.4730; *p* = 0.6271, using the Friedman Test). As such, this experiment, which was designed to explore the possibility that neurons’ inherent sensitivity is linked to their habituation properties, has failed to find evidence of such a link. Indeed, the results suggest that the visual neurons’ habituation properties are very similar regardless of the stimulus properties that they detect.

## Discussion

In this study, we have used brain-wide calcium imaging to identify the response profiles and locations of visual neurons across the larval zebrafish brain. Focusing on loom stimuli and the luminance changes that compose them, we have echoed previous results finding similar distribution of responses across the brain (Dunn et al., 2016; Yao et al., 2016; Cheng et al., 2017; Zhang et al., 2017; Chen et al., 2018; Heap L. A. L. et al., 2018; Helmbrecht et al., 2018; Marquez-Legorreta et al., 2019; Fernandes et al., 2021; Robles et al., 2021). A new interesting finding is that responses to motion are numerous and widespread, while responses to changes in luminance are more localized. The widespread response to checkerboard looms, with similar distribution to looms, and the fact that checkerboard looms produce escape responses while dim stimuli do not, suggest that the perception of expanding edges is a key feature for the detection of possible threats. As such, this stimulus recruits a broad network of neurons to elicit a motor response, but also extensive upstream processing regions in addition to the core startle circuit. Although it has been shown that tectal cells are sensitive to directional movements (Gabriel et al., 2012; Hunter et al., 2013; Wang et al., 2019), how this information is combined for the perception of the expanding edges of a loom remains unclear. Another possible explanation for the spatial spread and prevalence of motion-sensing neurons is the wide range of ethologically relevant moving stimuli that larval zebrafish encounter, along with several critical behaviors that rely on motion perception. These behaviors include predation, the optomotor and optokinetic responses, social behaviors, and visual escape (Semmelhack et al., 2014; Dreosti et al., 2015; Naumann et al., 2016; Larsch and Baier, 2018; Henriques et al., 2019; Marquez-Legorreta et al., 2020; Wang et al., 2020), and while the visual stimuli eliciting these various responses are diverse, a large salient stimulus like a checkerboard loom might elicit responses from a broad swathe of visual perceptual circuits. We also note checkerboard responses in regions such as the thalamus, where we have previously failed to observe them (Heap L. A. L. et al., 2018). We account for this difference based on the current study’s volumetric imaging, functional segmentation (Giovannucci et al., 2019), and altered thresholds for inclusion or ROIs as responsive.

Although some evidence exists for loom-specific RGCs in larval zebrafish (Temizer et al., 2015), recent studies suggest that such RGCs tend to be also sensitive to other moving objects or dimming stimuli (Förster et al., 2020; Kölsch et al., 2021). In general, these previous studies find a different distribution of movement sensitive and dimming sensitive RGC axons in the tectal neuropil, suggesting that different RGCs process this information (Robles et al., 2014; Kölsch et al., 2021). Tectal cells, however, seem to be capable of computing new features based on the RGCs input (Hunter et al., 2013; Förster et al., 2020). We have shown that neurons that respond to both the motion and dimming components of loom stimuli are concentrated in the tectum (Figure 3). This supports the notion that the tectum (and likely the superior colliculus in mammals) is the first stage in the visual pathway at which loom stimuli are specifically encoded (Heap L. A. L. et al., 2018). Regardless of whether loom encoding is an emergent property of the tectum, these loom responses put the tectum in a likely position to perform or direct sensorimotor gating in the visual escape pathway (Yao et al., 2016; Helmbrecht et al., 2018).

Convergent visual information from the eye and thalamus, combined with processing integral to tectal circuits, could encode the presence, location, and speed of looming objects. The resulting sensorimotor gating could then be modulated in the tectum by inputs from a number of brain regions with information on the larva’s context and recent history (Filosa et al., 2016; Lovett-Barron et al., 2017; Heap L. A. et al., 2018; Yáñez et al., 2018; Henriques et al., 2019; Marques et al., 2019; Fernandes et al., 2021). Outputs from the tectum to downstream premotor neurons, therefore, could direct appropriate responses, depending on the details of the loom stimulus and the animal’s circumstances. In all of these regards, the results presented here fit in with a model of the tectum as a key pivot point in the visual escape pathway (Marquez-Legorreta et al., 2020; Isa et al., 2021).

Another goal of this study was to explore the relationships that exist between neurons’ sensitivity to the individual components of loom stimuli and their possible roles in habituation. Behaviorally, we found that checkerboard stimuli elicited escapes responses, but that these responses did not habituate after repeated checkerboard stimuli, as occurs during trains of repeated loom stimuli. Furthermore, we found that neither repeated checkerboard stimuli nor repeated dim stimuli pre-habituated larvae to subsequent looms, which elicited escape responses like those seen in naïve animals exposed to looms. As such, at least at the behavioral level, there is no evidence for habituation in movement-sensitive escape circuits, nor is there evidence for contributions to loom habituation from either motion-specific or dim-specific pathways. This suggests that both movement and luminance properties of loom stimuli are required for habituation of the loom response.

Using calcium imaging, we next explored whether neurons across the brain that respond to specific stimuli have characteristic habituation profiles. We found that all three functional categories that we identified (movement-responsive, luminance-responsive, and responsive to both) underwent habituation to repeated presentations of the relevant stimuli (checkerboards, dims, and full looms, respectively). While this was expected for full looms, which habituate behaviorally, it was more surprising for checkerboards, since there is no behavioral habituation to this stimulus (Figure 1). This uncoupling of our calcium imaging and behavioral data could result from the abundance and breadth of motion-sensitive neurons across the brain (discussed above), many of which may be involved in behaviors other than escape. This difference could be due to the stimulus’ always coming from the same visual location in the calcium imaging experiments, while in free-swimming larvae the visual stimuli varied depending on the position and orientation of the fish. The role of visual position, and the consistency of visual position in repeated loom stimuli, would be an interesting future avenue to pursue. Another feature that could explain habituation in the neuronal responses while not in the free-swimming behavior is the lack of sensory feedback after an attempted escape response. The lack of this feedback could affect the fish’s processing of the stimulus and facilitate habituation, as such absence of feedback has been shown to induce passivity (Mu et al., 2019). Finally, we explored the possibility that neurons with specific response profiles (to loom components) would have characteristic and differing habituation rates, based on similarities seen between these neurons’ distributions in this study and the distributions of neurons with different habituation rates from a prior study (Marquez-Legorreta et al., 2019). Refuting this possibility, we found that motion-sensitive neurons, dim-sensitive neurons, and neurons responsive to both components all had similar habituation profiles in animals presented with repeated loom stimuli.

The cellular and sub-cellular mechanisms underlying behavioral habituation are complex (reviewed in McDiarmid et al., 2019). As we have shown, the relationship between the amplitude of the response from stimulus-sensitive neurons and the probability of a behavioral response is not linear. We have shown habituation in responses at the cellular level to all three stimuli used. On the timescales that we are observing, this is more likely to result from reduction in neurotransmitter release at the presynaptic site than postsynaptic mechanisms (Castellucci and Kandel, 1974; Glanzman, 2009). However, we observe habituation across all the responsive structures in the brain, so the spatial origin of the decreases and the ways in which these attenuated responses are propagated through the brain are not clear. Importantly, we have shown that habituation to one component of the loom (either moving edges or dimming), does not generalize to habituate responses across the network of neurons responsive to the full loom.

Overall, the outcomes of this study, while incremental, shed light on specific details of the visual escape pathway and its habituation. Behaviorally, we show that escape responses only habituate during repeated full loom stimuli, and that neither the movement nor the luminance components of loom stimuli contribute to behavioral loom habituation by themselves. Our brain-wide calcium imaging shows broader and more numerous responses to movement than to dimming stimuli, and provides no evidence for loom-specific neurons (that is, neurons that respond to loom but no to movement or dimming alone). Furthermore, these results show that neurons responsive to both movement and dimming stimuli are concentrated in the tectum, further suggesting an important role for this structure in detecting and responding to threatening visual stimuli. Finally, we have failed to find a link between neurons’ initial response selectivity and habituation rate during repeated looms. Such relationships would have suggested that the processing of particular stimulus properties plays specific roles in brain-wide visual habituation. The absence of such relationships suggests that habituation may rest with local microcircuits in the tectum or in interactions across multiple functional brain-wide populations of neurons. The detailed mechanisms of these local or brain-wide networks provide a rich topic for future exploration.

## Data Availability Statement

The original contributions presented in the study are available at: https://doi.org/10.48610/3684089.

## Ethics Statement

The animal study was reviewed and approved by the University of Queensland Animal Welfare Unit under approval SBS/341/19.

## Author Contributions

TM collected and analyzed data and wrote the manuscript. EM-L designed experiments, collected data, performed analyses, and wrote the manuscript. MW guided experimental analysis and wrote the manuscript. MP collected and analyzed data. IF-B designed, built, maintained the light-sheet microscope, and edited the manuscript. GV performed analyses and edited the manuscript. ES designed experiments, guided analyses, and wrote the manuscript. All authors contributed to the article and approved the submitted version.

## Author Disclaimer

The content is solely the responsibility of the authors and does not necessarily represent the official views of the National Institutes of Health.

## Conflict of Interest

The authors declare that the research was conducted in the absence of any commercial or financial relationships that could be construed as a potential conflict of interest.

## Publisher’s Note

All claims expressed in this article are solely those of the authors and do not necessarily represent those of their affiliated organizations, or those of the publisher, the editors and the reviewers. Any product that may be evaluated in this article, or claim that may be made by its manufacturer, is not guaranteed or endorsed by the publisher.

## Footnotes

1
http://github.com/flatironinstitute/CaImAn

2
https://github.com/ANTsX/ANTs

## References

[B1] Bhattacharyya K. McLean D. MacIver M. (2017). Visual threat assessment and reticulospinal encoding of calibrated responses in larval zebrafish. *Curr. Biol.* 27 2751–2762.e6. 10.1016/j.cub.2017.08.012 28889979 PMC5703209

[B2] Burgess H. A. Granato M. (2007). Sensorimotor gating in larval zebrafish. *J. Neurosci.* 27 4984–4994. 10.1523/jneurosci.0615-07.2007 17475807 PMC6672105

[B3] Castellucci V. F. Kandel E. R. (1974). Quantal analysis of synaptic depression underlying habituation of gill-withdrawal reflex in *Aplysia*. *Proc. Natl. Acad. Sci. U.S.A.* 71 5004–5008. 10.1073/pnas.71.12.5004 4373738 PMC434028

[B4] Chen T.-W. Wardill T. J. Sun Y. Pulver S. R. Renninger S. L. Baohan A. (2013). Ultrasensitive fluorescent proteins for imaging neuronal activity. *Nature* 499 295–300. 10.1038/nature12354 23868258 PMC3777791

[B5] Chen X. Mu Y. Hu Y. Kuan A. T. Nikitchenko M. Randlett O. (2018). Brain-wide organization of neuronal activity and convergent sensorimotor transformations in larval zebrafish. *Neuron* 100 876–890.e5. 10.1016/j.neuron.2018.09.042 30473013 PMC6543271

[B6] Cheng R.-K. Krishnan S. Lin Q. Kibat C. Jesuthasan S. (2017). Characterization of a thalamic nucleus mediating habenula responses to changes in ambient illumination. *BMC Biol.* 15:104. 10.1186/S12915-017-0431-1 29100543 PMC5670518

[B7] Constantin L. Poulsen R. E. Scholz L. A. Favre-Bulle I. A. Taylor M. A. Sun B. (2020). Altered brain-wide auditory networks in a zebrafish model of fragile X syndrome. *BMC Biol.* 18:125. 10.1186/s12915-020-00857-6 32938458 PMC7493858

[B8] Douglass A. D. Kraves S. Deisseroth K. Schier A. F. Engert F. (2008). Escape behavior elicited by single, channelrhodopsin-2-evoked spikes in zebrafish somatosensory neurons. *Curr. Biol.* 18 1133–1137. 10.1016/J.CUB.2008.06.077 18682213 PMC2891506

[B9] Dreosti E. Lopes G. Kampff A. R. Wilson S. W. (2015). Development of social behavior in young zebrafish. *Front. Neural Circuits* 9:39. 10.3389/Fncir.2015.00039 26347614 PMC4539524

[B10] Dunn T. W. Gebhardt C. Naumann E. A. Riegler C. Ahrens M. B. Engert F. (2016). Neural circuits underlying visually evoked escapes in larval zebrafish. *Neuron* 89 613–628. 10.1016/j.neuron.2015.12.021 26804997 PMC4742414

[B11] Eaton R. Farley R. Kimmel C. Schabtach E. (1977). Functional development in the Mauthner cell system of embryos and larvae of the zebra fish. *J. Neurobiol.* 8 151–172. 10.1002/neu.480080207 856948

[B12] Favre-Bulle I. A. Taylor M. A. Marquez-Legorreta E. Vanwalleghem G. Poulsen R. E. Rubinsztein-Dunlop H. (2020). Sound generation in zebrafish with Bio-Opto-Acoustics (BOA). *bioRxiv* [Preprint]. 10.1101/2020.06.09.143362 PMC7705743 33257652

[B13] Favre-Bulle I. A. Vanwalleghem G. Taylor M. A. Rubinsztein-Dunlop H. Scott E. K. (2018). Cellular-resolution imaging of vestibular processing across the larval zebrafish brain. *Curr. Biol.* 28 3711–3722.e3. 10.1016/j.cub.2018.09.060 30449665

[B14] Fernandes A. M. Mearns D. S. Donovan J. C. Larsch J. Helmbrecht T. O. Kölsch Y. (2021). Neural circuitry for stimulus selection in the zebrafish visual system. *Neuron* 109 805–822.e6. 10.1016/j.neuron.2020.12.002 33357384

[B15] Filosa A. Barker A. J. Dal Maschio M. Baier H. (2016). Feeding state modulates behavioral choice and processing of prey stimuli in the zebrafish tectum. *Neuron* 90 596–608. 10.1016/j.neuron.2016.03.014 27146269

[B16] Förster D. Helmbrecht T. O. Mearns D. S. Jordan L. Mokayes N. Baier H. (2020). Retinotectal circuitry of larval zebrafish is adapted to detection and pursuit of prey. *Elife* 9:e58596. 10.7554/elife.58596 33044168 PMC7550190

[B17] Gabriel J. Trivedi C. Maurer C. Ryu S. Bollmann J. (2012). Layer-specific targeting of direction-selective neurons in the zebrafish optic tectum. *Neuron* 76 1147–1160. 10.1016/j.neuron.2012.12.003 23259950

[B18] Giovannucci A. Friedrich J. Gunn P. Kalfon J. Brown B. L. Koay S. A. (2019). CaImAn an open source tool for scalable calcium imaging data analysis. *Elife* 8:e38173. 10.7554/eLife.38173 30652683 PMC6342523

[B19] Glanzman D. L. (2009). Habituation in *Aplysia*: the cheshire cat of neurobiology. *Neurobiol. Learn. Mem.* 92 147–154. 10.1016/j.nlm.2009.03.005 19332142

[B20] Heap L. A. L. Vanwalleghem G. Thompson A. W. Favre-Bulle I. A. Scott E. K. (2018). Luminance changes drive directional startle through a thalamic pathway. *Neuron* 99 293–301.e4. 10.1016/j.neuron.2018.06.013 29983325

[B21] Heap L. A. Vanwalleghem G. C. Thompson A. W. Favre-Bulle I. Rubinsztein-Dunlop H. Scott E. K. (2018). Hypothalamic projections to the optic tectum in larval zebrafish. *Front. Neuroanat.* 11:135. 10.3389/fnana.2017.00135 29403362 PMC5777135

[B22] Helmbrecht T. O. dal Maschio M. Donovan J. C. Koutsouli S. Baier H. (2018). Topography of a visuomotor transformation. *Neuron* 100 1429–1445.e4. 10.1016/j.neuron.2018.10.021 30392799

[B23] Henriques P. M. Rahman N. Jackson S. E. Bianco I. H. (2019). Nucleus isthmi is required to sustain target pursuit during visually guided prey-catching. *Curr. Biol.* 29 1771–1786.e5. 10.1016/j.cub.2019.04.064 31104935 PMC6557330

[B24] Hunter P. R. Lowe A. S. Thompson I. D. Meyer M. P. (2013). Emergent properties of the optic tectum revealed by population analysis of direction and orientation selectivity. *J. Neurosci.* 33 13940–13945. 10.1523/jneurosci.1493-13.2013 23986231 PMC3756745

[B25] Isa T. Marquez-Legorreta E. Grillner S. Scott E. K. (2021). The tectum/superior colliculus as the vertebrate solution for spatial sensory integration and action. *Curr. Biol.* 31 R741–R762. 10.1016/j.cub.2021.04.001 34102128 PMC8190998

[B26] Kölsch Y. Hahn J. Sappington A. Stemmer M. Fernandes A. M. Helmbrecht T. O. (2021). Molecular classification of zebrafish retinal ganglion cells links genes to cell types to behavior. *Neuron* 109 645–662.e9. 10.1016/j.neuron.2020.12.003 33357413 PMC7897282

[B27] Larsch J. Baier H. (2018). Biological motion as an innate perceptual mechanism driving social affiliation. *Curr. Biol.* 28 3523–3532.e4. 10.1016/j.cub.2018.09.014 30393036

[B28] Lovett-Barron M. Andalman A. S. Allen W. E. Vesuna S. Kauvar I. Burns V. M. (2017). Ancestral circuits for the coordinated modulation of brain state. *Cell* 171 1411–1423.e17. 10.1016/j.cell.2017.10.021 29103613 PMC5725395

[B29] Luca R. Gerlai R. (2012). In search of optimal fear inducing stimuli: differential behavioral responses to computer animated images in zebrafish. *Behav. Brain Res.* 226 66–76. 10.1016/j.bbr.2011.09.001 21920389 PMC3203217

[B30] Marques J. C. Lackner S. Félix R. Orger M. B. (2018). Structure of the zebrafish locomotor repertoire revealed with unsupervised behavioral clustering. *Curr. Biol.* 28 181–195.e5. 10.1016/j.cub.2017.12.002 29307558

[B31] Marques J. C. Li M. Schaak D. Robson D. N. Li J. M. (2019). Internal state dynamics shape brainwide activity and foraging behaviour. *Nature* 577 239–243. 10.1038/s41586-019-1858-z 31853063

[B32] Marquez-Legorreta E. Constantin L. Piber M. Favre-Bulle I. A. Taylor M. A. Vanwalleghem G. C. (2019). Brain-wide visual habituation networks in wild type and fmr1 zebrafish. *bioRxiv* [Preprint]. 10.1101/722074 PMC8850451 35173170

[B33] Marquez-Legorreta E. Piber M. Scott E. K. (2020). “Visual escape in larval zebrafish: stimuli, circuits, and behavior,” in *Behavioral and Neural Genetics of Zebrafish*, ed. Gerlai R. T. (Amsterdam: Elsevier), 49–71. 10.1016/b978-0-12-817528-6.00004-8

[B34] McDiarmid T. A. Yu A. J. Rankin C. H. (2019). Habituation is more than learning to ignore: multiple mechanisms serve to facilitate shifts in behavioral strategy. *BioEssays* 41:e1900077. 10.1002/bies.201900077 31429094

[B35] McHenry M. J. Feitl K. E. Strother J. A. Trump W. J. V. (2009). Larval zebrafish rapidly sense the water flow of a predator’s strike. *Biol. Lett.* 5 477–479. 10.1098/rsbl.2009.0048 19324627 PMC2781903

[B36] Mu Y. Bennett D. V. Rubinov M. Narayan S. Yang C. T. Tanimoto M. (2019). Glia accumulate evidence that actions are futile and suppress unsuccessful behavior. *Cell* 178 27–43.e19. 10.1016/j.cell.2019.05.050 31230713

[B37] Naumann E. Fitzgerald J. Dunn T. Rihel J. Sompolinsky H. Engert F. (2016). From whole-brain data to functional circuit models: the zebrafish optomotor response. *Cell* 167 947–960.e20. 10.1016/j.cell.2016.10.019 27814522 PMC5111816

[B38] Noguchi K. Gel Y. R. Brunner E. Konietschke F. (2012). nparLD: an R software package for the nonparametric analysis of longitudinal data in factorial experiments. *J. Stat. Softw.* 50 1–23. 10.18637/jss.V050.I1225317082

[B39] Pnevmatikakis E. A. Giovannucci A. (2017). NoRMCorre: an online algorithm for piecewise rigid motion correction of calcium imaging data. *J. Neurosci. Methods* 291 83–94. 10.1016/j.jneumeth.2017.07.031 28782629

[B40] Pnevmatikakis E. A. Soudry D. Gao Y. Machado T. A. Merel J. Pfau D. (2016). Simultaneous denoising, deconvolution, and demixing of calcium imaging data. *Neuron* 89 285–299. 10.1016/j.neuron.2015.11.037 26774160 PMC4881387

[B41] Poulsen R. E. Scholz L. A. Constantin L. Favre-Bulle I. Vanwalleghem G. C. Scott E. K. (2021). Broad frequency sensitivity and complex neural coding in the larval zebrafish auditory system. *Curr. Biol.* 31 1977–1987.e4. 10.1016/j.cub.2021.01.103 33657408 PMC8443405

[B42] Randlett O. Haesemeyer M. Forkin G. Shoenhard H. Schier A. F. Engert F. (2019). Distributed plasticity drives visual habituation learning in larval zebrafish. *Curr. Biol.* 29 1337–1345.e4. 10.1016/j.cub.2019.02.039 30955936 PMC6545104

[B43] Randlett O. Wee C. L. Naumann E. A. Nnaemeka O. Schoppik D. Fitzgerald J. E. (2015). Whole-brain activity mapping onto a zebrafish brain atlas. *Nat. Methods* 12 1039–1046. 10.1038/Nmeth.3581 26778924 PMC4710481

[B44] Rankin C. H. Abrams T. Barry R. J. Bhatnagar S. Clayton D. F. Colombo J. (2009). Habituation revisited: an updated and revised description of the behavioral characteristics of habituation. *Neurobiol. Learn. Mem.* 92 135–138. 10.1016/j.nlm.2008.09.012 18854219 PMC2754195

[B45] Robles E. Fields N. P. Baier H. (2021). The zebrafish visual system transmits dimming information via multiple segregated pathways. *J. Comp. Neurol.* 529 539–552. 10.1002/cne.24964 32484919

[B46] Robles E. Laurell E. Baier H. (2014). The retinal projectome reveals brain-area-specific visual representations generated by ganglion cell diversity. *Curr. Biol.* 24 2085–2096. 10.1016/j.cub.2014.07.080 25155513

[B47] Sato T. Hamaoka T. Aizawa H. Hosoya T. Okamoto H. (2007). Genetic single-cell mosaic analysis implicates ephrinB2 reverse signaling in projections from the posterior tectum to the hindbrain in zebrafish. *J. Neurosci.* 27 5271–5279. 10.1523/jneurosci.0883-07.2007 17507550 PMC6672335

[B48] Semmelhack J. L. Donovan J. C. Thiele T. R. Kuehn E. Laurell E. Baier H. (2014). A dedicated visual pathway for prey detection in larval zebrafish. *Elife* 3:e04878. 10.7554/elife.04878 25490154 PMC4281881

[B49] Taylor M. A. Vanwalleghem G. C. Favre-Bulle I. A. Scott E. K. (2018). Diffuse light-sheet microscopy for stripe-free calcium imaging of neural populations. *J. Biophotonics* 11:e201800088. 10.1002/jbio.201800088 29920963

[B50] Temizer I. Donovan J. C. Baier H. Semmelhack J. L. (2015). A visual pathway for looming-evoked escape in larval zebrafish. *Curr. Biol.* 25 1823–1834. 10.1016/j.cub.2015.06.002 26119746

[B51] Thompson A. W. Scott E. K. (2016). Characterisation of sensitivity and orientation tuning for visually responsive ensembles in the zebrafish tectum. *Sci. Rep.* 61:34887. 10.1038/srep34887 27713561 PMC5054398

[B52] Thompson A. W. Vanwalleghem G. C. Heap L. A. Scott E. K. (2016). Functional profiles of visual-, auditory-, and water flow-responsive neurons in the zebrafish tectum. *Curr. Biol.* 26 743–754. 10.1016/j.cub.2016.01.041 26923787

[B53] Thompson R. F. Spencer W. A. (1966). Habituation: a model phenomenon for the study of neuronal substrates of behavior. *Psychol. Rev.* 73 16–43. 10.1037/h0022681 5324565

[B54] Vanwalleghem G. Constantin L. Scott E. K. (2021). Calcium imaging and the curse of negativity. *Front. Neural Circuits* 14:607391. 10.3389/fncir.2020.607391 33488363 PMC7815594

[B55] Wang K. Hinz J. Haikala V. Reiff D. F. Arrenberg A. B. (2019). Selective processing of all rotational and translational optic flow directions in the zebrafish pretectum and tectum. *BMC Biol.* 171:29. 10.1186/S12915-019-0648-2 30925897 PMC6441171

[B56] Wang K. Hinz J. Zhang Y. Thiele T. R. Arrenberg A. B. (2020). Parallel channels for motion feature extraction in the pretectum and tectum of larval zebrafish. *Cell Rep.* 30 442–453.e6. 10.1016/j.celrep.2019.12.031 31940488

[B57] Yáñez J. Suárez T. Quelle A. Folgueira M. Anadón R. (2018). Neural connections of the pretectum in zebrafish (Danio rerio). *J. Comp. Neurol.* 526 1017–1040. 10.1002/cne.24388 29292495

[B58] Yao Y. Li X. Zhang B. Yin C. Liu Y. Chen W. (2016). Visual cue-discriminative dopaminergic control of visuomotor transformation and behavior selection. *Neuron* 89 598–612. 10.1016/j.neuron.2015.12.036 26804989

[B59] Zhang B. Yao Y. Zhang H. Kawakumi K. Du J. (2017). Left habenula mediates light-preference behavior in zebrafish via an asymmetrical visual pathway. *Neuron* 93 914–928.e4. 10.1016/j.neuron.2017.01.011 28190643

