# Supplementary material for: Contributions of Luminance and Motion to Visual Escape and Habituation in Larval Zebrafish
Source: Front Neural Circuits. 2021 Oct 21;15:748535. doi: 10.3389/fncir.2021.748535 (PMC8568047; doi:10.3389/fncir.2021.748535)
Supplement: Supplementary file 1 [file Data_Sheet_1.PDF]

Supplementary Information for:

Contributions of luminance and motion to visual escape and habituation in larval zebrafish

Tessa Mancienne, Emmanuel Marquez-Legorreta, Maya Wilde, Marielle Piber, Itia Favre-Bulle, Gilles Vanwalleghem, and Ethan K. Scott

Supplementary Table 1: Thresholds used to categorize ROIs

**Response profile**	**Threshold**
Dim sensitive	max response to the first two dims > 1SD of all ROIs dim responses<br>AND<br>max response to the first two checkerboards < 1SD of all ROIs checkerboard responses
Checkerboard sensitive	max response to the first two checkerboards > 1SD of all ROIs checkerboard responses<br>AND<br>max response to the first two dims < 1SD of all ROIs dim responses
Dim + checkerboard sensitive	max response to the first two dims > 1SD of all ROIs dim responses<br>AND<br>max response to first the two checkerboards > 1SD of all ROIs checkerboard responses

Supplementary Table 1: Thresholds used in the analysis of the third SPIM dataset. ROIs were classified as having the response profiles defined in the left column if the amplitude of their responses to the first four stimuli met the criteria in the right column.

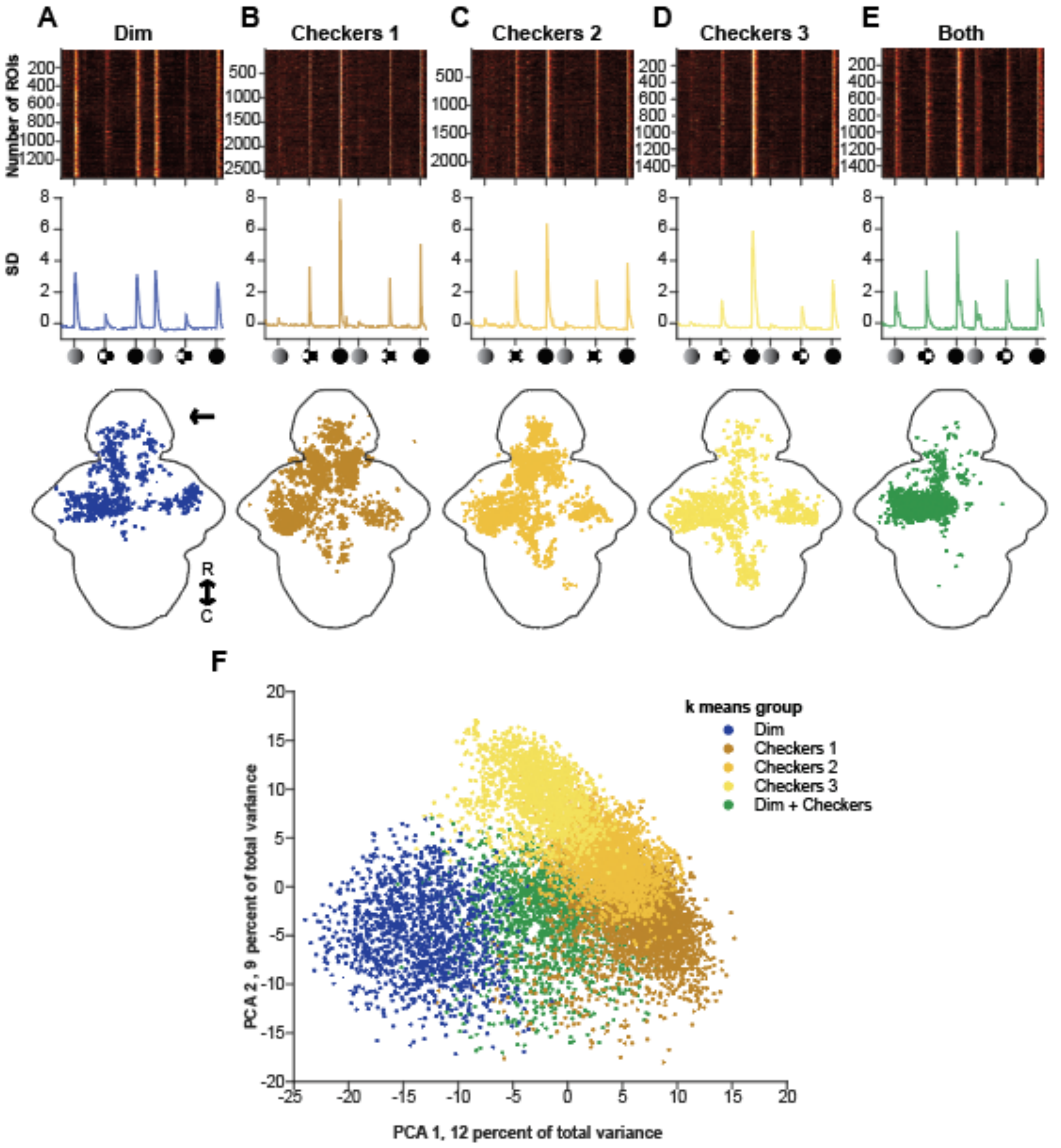

Supplemental Figure 1: Unmerged clusters from K-mean clustering performed on the second calcium dataset.

(A) A raster plot (top), average trace (middle), and anatomical position (bottom) of the dim sensitive (Dim) ROI cluster, arrow represents position of stimulus presentation. (B-D) show the same information for the three clusters that were sensitive to checkerboards (Checkers), and (E) shows this information for the ROI cluster sensitive to both dims and checkerboards (Both). (F) A principal component analysis run on the five clusters shows the three checkerboard-responsive clusters lie along a continuum of responses. R, rostral; C, caudal.

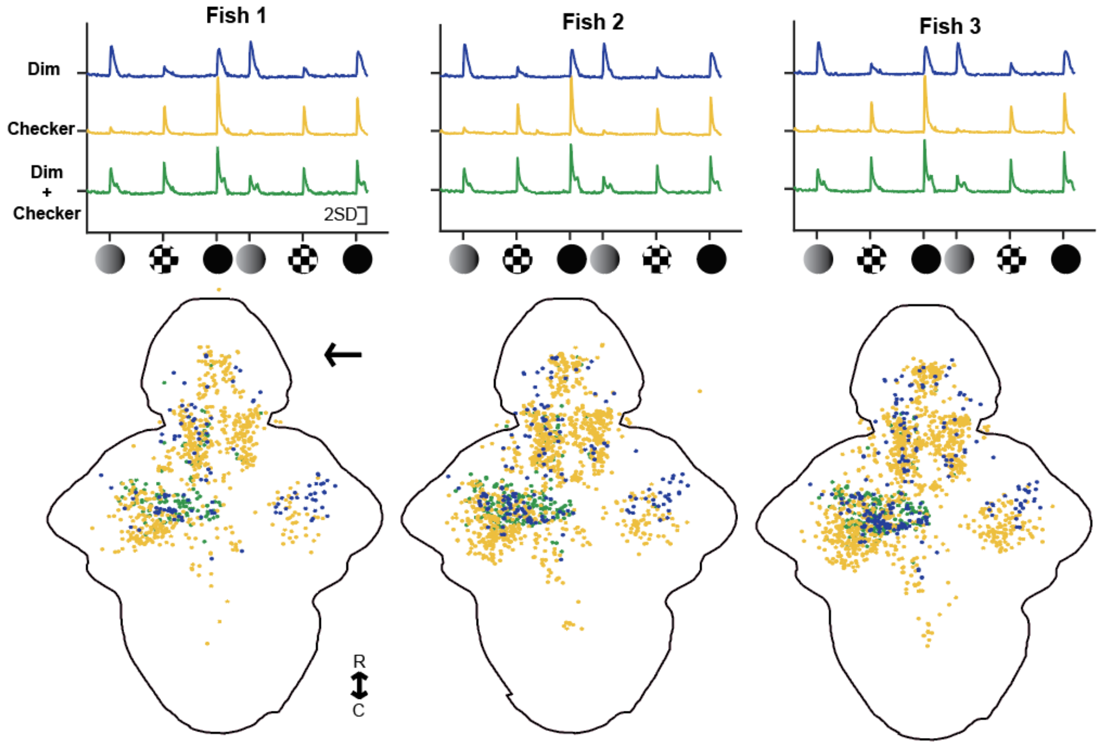

Supplemental Figure 2: Consistency of responses across individual fish.
Average trace (top) and anatomical position (bottom) of each of the clusters identified in the second calcium dataset for three fish, shown individually. R, rostral; C, caudal; arrow represents position of stimulus presentation.

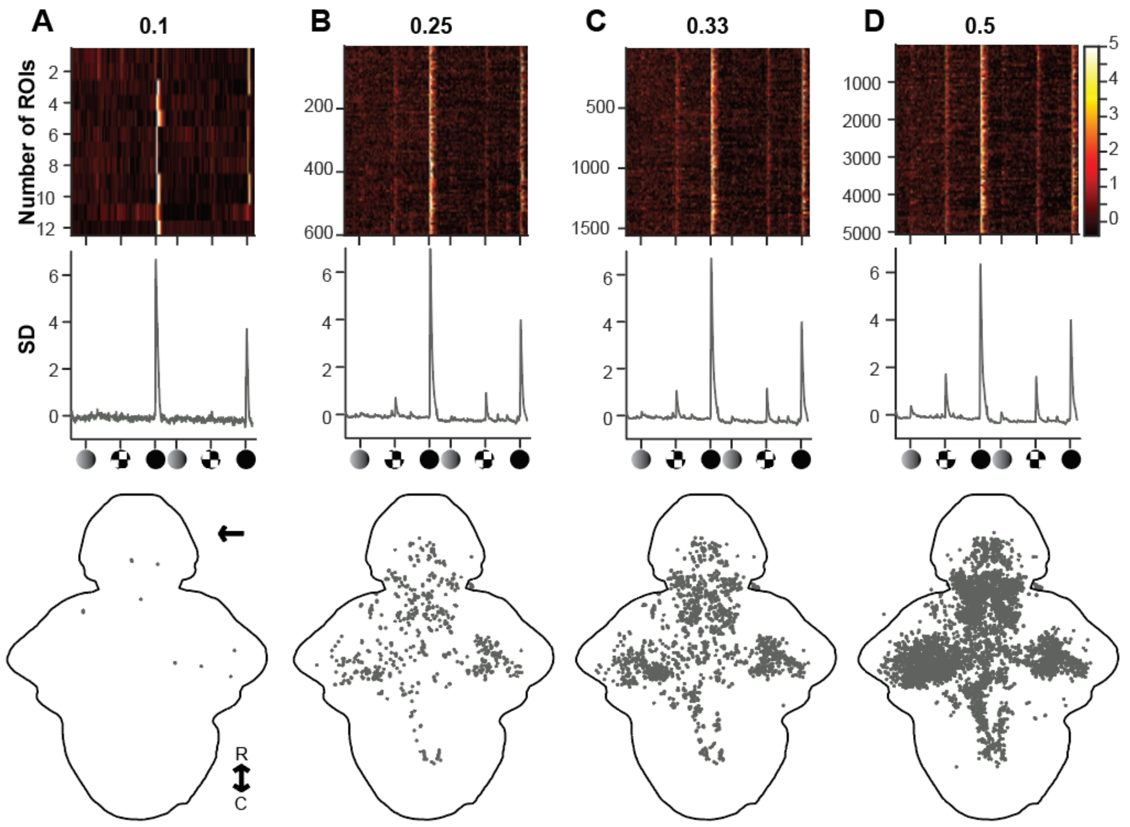

<u>**Supplemental Figure 3: Searching for loom-exclusive ROIs.**</u>

(A) A raster plot (top), average trace (middle), and anatomical position (bottom) of ROIs with a maximum loom response 10 times greater (dim/check:loom ratio < 0.1) than either dim or checkerboard responses. (B) shows the same information for loom responses 4 times greater (dim/check:loom ratio < 0.25) than either dim or checkerboard, (C) ROIs with maximum loom response 3 times greater (dim/check:loom ratio < 0.33) than either dim or checkerboard, (D) ROIs with maximum loom response 2 times greater (dim/check:loom ratio < 0.5) than either dim or checkerboard responses. R, rostral; C, caudal; arrow represents position of stimulus presentation.

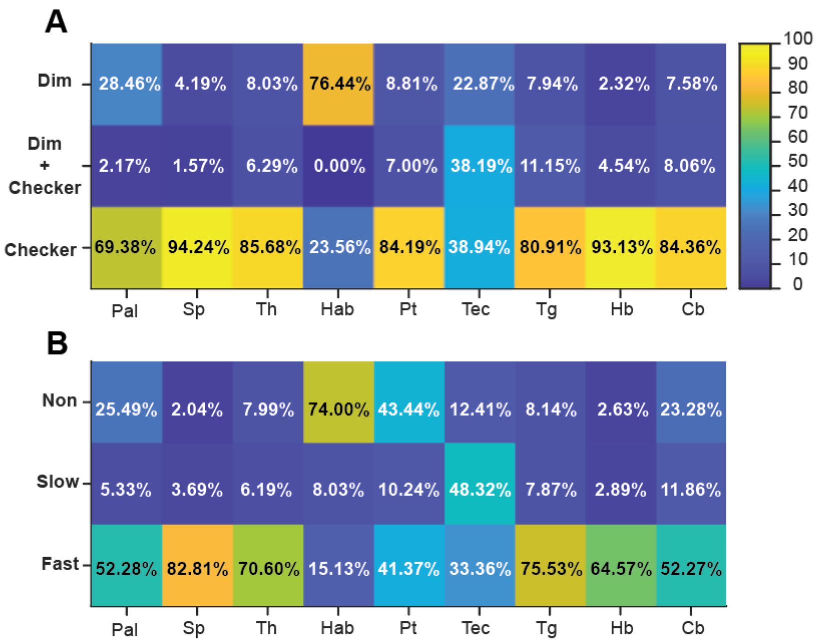

Supplemental Figure 4: Comparison between the identified component-sensitive clusters and the different rates of habituation.

(A) heatmap of normalized proportions of ROIs within each brain region that belong to the dim-sensitive, checkerboard-sensitive and the both (dim and checkerboard)-sensitive groups. Percentages are of the total number of visually responsive ROIs in the brain region. (B) heatmap of normalized proportions of loom responsive ROIs in each brain region for the non-habituating (non), slowly-habituating (slow) and rapidly-habituating (fast) ROIs in the dataset of (Marquez-Legorreta et al. (2019) Brain-wide visual habituation networks in wild type and fmr1 zebrafish. *bioRxiv*, 722074. doi:10.1101/722074). Loom responsive ROIs that strongly correlated to motor output are excluded from the heatmap. Pal, pallium; Sp, subpallium; Th, thalamus; Hab, habenula; Pt, pretectum; Tec, tectum; Tg, tegmentum; Hb, hindbrain; Cb, cerebellum.

Supplementary methods

Analysis of whole brain calcium activity

In the second SPIM dataset, our K-means group selection criteria resulted in 5 clusters which were well represented across fish. Supplementary Figure 1A-E shows raster plots and anatomical locations of these clusters. The three checkerboard-responsive clusters were merged manually based on their showing specificity to the checkerboard stimulus, however we also performed a principal component analysis (PCA) in Matlab 2018b (Statistics and Machine Learning Toolbox) on the response traces of all the included ROIs in order to visualize relationships and similarity among the K-means clusters. The first two PCAs represented approximately 21% of the total variance of ROI activity. The included ROIs were plotted across the first two principle components in Supplementary Figure 1F, which suggest that the dim responsive and checkerboard responsive ROIs are located on either side of the ROIs which respond to both stimuli, and the three checkerboard responsive clusters exist on a continuum.

In the second dataset, used to identify ROIs that responded preferentially to checkerboard or dim stimuli (or both), no ROIs selectively responding only to full looms emerged from the K-means analysis. To actively search for potential loom-specific ROIs amongst those that were visually responsive, for each ROI we found the maximum amplitude response across all the dim and checkerboard presentations and divided this by the maximum response during the loom presentations. We then classified ROIs with loom responses 10, 5, 3 and 2 times greater than any dim or check response (Supplementary Figure 3).

The spatial distribution of the ROIs which preferentially responded to either dim or checkerboard components of the loom appeared similar to the distribution of populations of ROIs that habituate to full looms at different rates, as shown in a previous paper (Marquez-Legorreta et al. (2019) Brain-wide visual habituation networks in wild type and fmr1 zebrafish. *bioRxiv*, 722074. doi:10.1101/722074). A breakdown of the brain regions contributing to each group can be found in Supplementary Figure 4A. For comparison to the distributions of rapidly habituating, slowly habituating and non-habituating groups of ROIs from the previous paper, the distribution of ROIs from those groups has been included in Supplementary Figure 4B. The similarity between these distributions formed the basis for the third calcium imaging dataset which compared component specificity and loom habituation rate.

The distribution and response profile of dim, checkerboard and both sensitive clusters were relatively consistent across fish, as can be observed in Supplementary Figure 2.
